# Combined Effects of High-Intensity Aerobic Exercise Training and *Ziziphus jujuba* Extract on Tissue Nesfatin-1 in Rats

**DOI:** 10.3389/fendo.2022.845014

**Published:** 2022-05-03

**Authors:** Abbass Ghanbari-Niaki, Fahimeh Hosseini, David Robert Broom, Bahareh Tejenjari, Saleh Rahmati-Ahmadabad

**Affiliations:** ^1^ Exercise Biochemistry Division, Faculty of Sport Sciences, University of Mazandaran, Babolsar, Iran; ^2^ Centre for Sport, Exercise and Life Sciences, Coventry University, Coventry, United Kingdom; ^3^ Department of Physical Education, Pardis Branch, Islamic Azad University, Pardis, Iran

**Keywords:** exercise, cardiovascular disease, nestafin-1, supplementation, *Ziziphus jujuba*

## Abstract

Nesfatin-1 is involved in metabolic/feeding regulation and prevention of cardiovascular disease. Previous studies have shown that exercise and herb supplementation can influence nesfatin-1 concentration. The present study investigated the effects of high-intensity training (HIT) and Ziziphus jujuba (ZJ) extract on tissue nesfatin-1 in rats. Twenty-eight female rats were randomly assigned to one of four groups i.e. 1) Saline-Control (SC), 2) Saline-High Intensity Training (ST), 3) Ziziphus jujuba-Control (ZJC), and 4) Ziziphus jujuba-High Intensity Training (ZJT). Rats performed exercise on a treadmill and/or administered supplements intragastrically for 6 weeks, depending on group category. Seventy-two hours after the last training session, rats were anesthetized. Blood, hypothafi 2lamus tissue, heart and gastrocnemius muscles were sent to the laboratory for analyses. Significantly higher nesfatin-1 gene expression and concentration and ATP concentration were found in trained rat. HIT increased plasma High Density Lipoprotein (HDL) and insulin concentration and reduced plasma Triglyceride (TG) and cortisol. ZJ increased tissue nesftain-1 gene expression and concentration while only increasing heart ATP. The combination of exercise and ZJ showed an additive effect compared to each intervention alone on hypothalamus, heart and gastrocnemius NUCB2 gene expression, heart and gastrocnemius nesfatin-1 concentration, plasma HDL and cortisol concentration. The authors recommend both interventions as a means to improve cardiovascular health in rats with further work needed to confirm similar findings in homo sapiens.

## Introduction

Nesfatin-1 is a neuropeptide involved in metabolic regulation and feeding behavior (1). Studies have highlighted that plasma nesfatin-1 and its gene expression changes can influence the cardiovascular system and its functions and related elements including high-density lipoprotein (HDL), low-density lipoprotein (LDL), triglycerides (TG), and total cholesterol (TC) (1). Nesfatin-1 is an anti-hyperglycemic, neuroendocrine regulator suppressor and is related to insulin and cortisol concentrations (2).

Nesfatin-1 has been detected in serum and plasma and is regulated by several factors including fasting (3), nutrient intake (4), steroid hormones (5), herb supplements [e.g. black cumin seeds (6) Nigella sativa (7) Pistacia Atlantica (8), Ziziphus jujuba (ZJ) (9)] and exercise (8–10).

In this study, we investigated two interventions being 1) physical activity by undertaking high intensity exercise training and 2) herb supplement use. It was hypothesized both could potentially change nesfatin-1 concentration with a combination having a more pronounced effect. Physical exercise has been shown to alter nesfatin-1 concentration in moderate (8), but not high intensity (9) aerobic exercise training (HIT). However, another study showed that high intensity training had more effect on nesfatin-1 compared to moderate intensity exercise (11). These discrepancies show that the intensity of exercise is an important determinant in influencing nesfatin-1 concentration and findings warrant confirmation (12).

Regarding supplement use, ZJ is a medicinal herb containing several constituents including water, protein, lipids, carbohydrates, vitamins and minerals and with no cholesterol contents (13). It has been reported to increase the concentration of muscle adenosine triphosphate (ATP) and glycogen content as two main energy sources at rest, during and after exercise (9). It has been shown that the crude extraction of ZJ resulted in a higher and significant increase in plasma and liver nesfatin-1 concentration in rats treated with ZJ. Results also indicate that an induced-ZJ nesfatin-1 elevation was accompanied with higher glycogen content in rat liver, while liver ATP concentration was unchanged (9). These findings have not been confirmed or refuted.

Ramanjaneya et al. (14) examined food deprivation for 24 hours on nucleobindin 2 (NUCB2) mRNA and nesfatin-1 protein expression compared to mice in the fed state. They reported that using RT-PCR and western blot techniques demonstrated that food deprivation (a factor reducing liver glycogen) resulted in a significant decrease in NUCB2 mRNA andnesfatin-1 protein expression levels in deprivated-micesubcutaneous adipose tissue when compared to fed mice (14). They also confirm that their findings are in agreement with Oh et al. who reported that NUCB2 gene expression was reduced in the paraventricular nucleus of hypothalamus, in which nesfatin-1 concentration was also decreased (15). Ramanjaneya et al. (14) suggests that NUCB2 mRNA and nesfatin-1 protein expression inmurine adipose tissue might be regulated by tissue energy supply. An elevation in tissue energy content would decrease food intake behavior exerting an anti-appetite effect *via* a higher plasma nesfatin-1 concentration and gene expression at different tissues, particularly in the brain and gastrointestinal tract in humans and rats (16, 17). It is suggested that performing high intensity exercise training on a treadmill as well as ZJ consumption has an effective role in the cardiac rehabilitation phase after a heart attack (18). Additionally, it is suggested that ZJ might suppress the apoptotic situation caused by exercise (19).

Based on the above rationale, the aim of the present study was to investigate the effects of six weeks HIT with or without ZJ supplementation on hypothalamus, heart and gastrocnemius nesfatin-1/NUCB2 gene expression, as well as, heart and gastrocnemius muscle nesfatin-1 concentration. In addition, the present study evaluates the relationship of nesfatin-1 concentration with cardiovascular risk factors (i.e. HDL, LDL, TG, and TC), energy sources (ATP and glycogen) and hormones (insulin, cortisol, and estradiol) in female rats.

## Materials and Methods

### Ethics

The present study was conducted according to the policy of the Iranian convention for the protection of vertebrate animals used for scientific experimental purposes and the National Institutes for Health guide for the care and use of laboratory animals (NIH publications no. 80–23). In addition, the protocol of the present study was approved by the ethics committee of the university of Mazandaran medical sciences (Code: IR.UMZ.REC.1400231).

### Animal Storage Conditions

Twenty-eight Wistar female rats (6-8 weeks old, 100-120 g body mass) were maintained in the Animal House (12-hour light-dark cycle, temperature: 22 (°C) ± 1.4 (°C) in which the floor was covered with wood chips. Animal standard compressed food was provided at regular times. Water was provided in 500 ml bottles that was attached to the animal cages.

### Animal Groups

Animals were randomly assigned into groups including Saline-Control (SC) (*n*=7), Saline-High Intensity Training (ST) (*n*=7), Ziziphus jujuba-Control (ZJC) (*n*=7), and Ziziphus jujuba-High Intensity Training (ZJT) (*n*=7). Animals performed exercise on a treadmill (0% grade, 35 m/min, 60 min/day, for 5 days/week), or administered supplements intragastrically (ZJ or saline solution equivalent to one mL/100g of body mass) for 6 weeks, depending on the group category. Supplements were administered intragastrically immediately after each exercise session or at the corresponding rest time.

### Sex Consideration

A high dose and chronic consumption of ZJ can reduce sex potential and libido in males (20) so we decided to examine female rats only.

Vaginal lavage observation was used to determine the estrous cycle each morning using a microscope. Female rats showing at least two consecutive 4- or 5-day estrous cycles were used in the present study (21).

### Preparation of *Ziziphus jujuba* Extract

Dried *Ziziphus jujuba* fruit was obtained from the main market of Birjand city (South Khorasan province, Iran), cleaned, washed with tap water, re-dried for 3 days in lab oven at 40°C. The herbalist of herbarium collection of the Biology department of Mazandaran University, Babolsar, Mazandaran, Iran identified the plant material. The seeds were removed and powdered by a house blender and the extraction was prepared according to Cacige et al. (22). Briefly, 40 mg of the powdered *Ziziphus jujuba* extracted with 600 ml water for 70 h at 10°C, followed by filtration. The final extract was 400 ml.

These procedures were chosen to prevent any possible increase in 5-hydroxymethylfurfural (HMF) which has been recognized as an end product of carbohydrate degradation when heating for *Ziziphus jujuba* extraction at different and high temperatures (23). According to Cacig et al. (22) who noticed that the preparation of *Ziziphus jujuba* extraction by maceration method at 10°C and four 70 hours did not show any mold growing as a result of fermentation.

Rats were orally gavaged by a liquid crude *Ziziphus* extract at dose 1g/kg of body weight (BW) (100 mg/100 g of BW) in 10 ml/kg of BW. The saline group was treated with the same volume of the normal saline as *Ziziphus* treated rats.

### Training Protocol

The rats were familiarized with the treadmill training protocol for 4 days prior to the study commencing after which there was a wash out period of 12 hours. The exercise groups were trained with a fixed intensity programme of treadmill running at 35 m/min for 60 minutes, 5 d/week for 6 weeks (9).

### Plasma Collection and Tissue Biopsies

Seventy-two hours after the last training session, rats were anesthetized with intraperitoneal administration of ketamine (30– 50 mg/kg body mass) and xylazine (3– 5 mg/kg body mass). Three hours before sacrifice, food but not water was removed. Following sacrifice blood was collected from the inferior vena cava close to the heart into EDTA test tubes. The test tubes were centrifuged for 10 min at 3000 rpm for plasma preparation. Hypothalamus tissue, heart and gastrocnemius muscles were excised, cleaned and washed with normal saline. All plasma and tissue samples were frozen in liquid nitrogen and stored at − 80 (°C) until laboratory analyses.

### Plasma and Tissue Measurements

A direct Immune method was used to determine plasma HDL concentration (HDL-C Immuno FS, Pars Azmoun, Tehran, Iran; Intraassay coefficient of variation: 1.2%, Interassay coefficient of variation sensitivity: 1.8%, sensitivity: 0.03 mmol/L). LDL-C was obtained according to the manufacturers’ protocol (an enzymatic method; kit was purchased from Pars Azma Iranian Com) (Intraassay coefficient of variation: 0.67%, Interassay coefficient of variation sensitivity: 1.45%, sensitivity: 2 mg/dL). Plasma insulin concentration was determined using a sandwich ELISA kit from ALPCO (Intraassay coefficient of variation: 3.4%, Interassay coefficient of variation sensitivity: 4.3%, sensitivity: 0.5 μIU/mL). An enzymatic (GPO, Glycerol-3-Phosphate Oxidase) colorimetric method (Pars Azmoun, Tehran, Iran; intra-assay coefficient of variation: 1.47%, Interassay coefficient of variation sensitivity: 1.06%, sensitivity: 1 mg/dL) was used to determine plasma total Triglyceride (TG). An enzymatic (CHOD-PAP, Cholesterol Oxidase-Amino Antipyrine) colorimetric method (Pars Azmoun, Tehran, Iran; intra-assay coefficient of variation: 1.9%, Interassay coefficient of variation sensitivity: 0.93%, sensitivity: 0.08 mmol/L) was used to determine plasma total cholesterol (TC). An ELISA method (Canada Inc, Ontario, Canada Estradiol ELISA, Diagnostics Biochem, intra-assay coefficient of variation: 4.6%, Interassay coefficient of variation sensitivity: 6.2%, sensitivity: 10 Pg/mL) was used to determine plasma estrogen concentration. Plasma cortisol also measured using Diagnostics Biochem ELISA kit (intra-assay coefficient of variation: 2.9%, Interassay coefficient of variation sensitivity: 3.8%, sensitivity: 0.4 μg/dL).

Tissue nesfatin-1 concentration was measured using a Rat Nesfatin-1 ELISA Kit (CUSABIO, Catalog No, CSB-E 1478r, China; sensitivity: 3.9 pg/mL, Intraassay: 7.5% and interassay <10%). A Bioluminescence method was used to determine tissue ATP concentration by special kit (BioVision Incorporated 155 S. Milpitas Boulevard, Milpitas, CA 95035 USA; quantitation range was approximately between 1 nmol and 10 fmol/assay). A Double Beam UV Spectrophotometer (Cecil Elegant Technology CE-5501 computing Cambridge England) was used to determine tissue glycogen concentration.

To measure the tissue NUCB2 gene expression, RNA was extracted by a specific kit (Cat. No. k3090, Bioneer, Daejeon, Republic of Korea) and all hypothalamic tissue and almost 100-mg of heart and gastrocnemius muscle were used. After that measure the purity of extracted RNA by spectrophotometer, cDNA was manufactured using AccuPower RT PreMix cDNA synthesis kit, Bioneer. The cDNA was kept at −20°C for use in real-time PCR. The real-time PCR was performed using Qiagen SYBR Green kit (one μl, Cat. No. 204052, Qiagen GmbH, Hilden, Germany), cDNA (1 μl) RNAse-free water (5 μl), forward and reverse primers (1 μl of each one) and using Australian Corbett’s machine. All stages were according to the Rahmati-Ahmadabad et al. article (24). The Threshold Cycle (CT) was recorded and converted to relative gene expression data using the 2^^- ΔΔCT^ method (24, 25). Unique primers for the real-time PCR stage were NUCB2 and GAPDH. The NUCB2 sense and antisense primers were 5’- TTTGAACACCTGAACCACCA-3’ and 5′- TGCAAACTTGGCTTCTTCCT-3′, respectively. The GAPDH sense and antisense primers were 5’- GTGCCAGCCTCGTCTCATAG-3’ and 5′- GACTGTGCCGTTGAACTTGC-3′, respectively.

### Statistical Analysis

All statistical analysis was undertaken using SPSS software, version 24 (SPSS, Inc., Chicago, IL). Descriptive statistics were used to categorize and determine the dispersion indices. The normality of data was determined using the Shapiro-Wilk test. Two-way ANOVA and the least significant difference (LSD) test was used to determine the significant changes between groups. Repeated measures two-way analysis of variance was used to evaluate the effects of time, group and time×group. If Mauchley’s test of sphericity was significant, Greenhouse–Geisser correction was used. Partial eta-squared ( $\eta_{\text{p}}^{2}$ ) is reported to emphasize the size of the difference rather than confound the sample size with 0.01, 0.03, and >0.05 considered small, medium, and large effects, respectively. Moreover, non-centrality parameter (NCP) and observed power/*post-hoc* power (OPW) were reported. The Pearson Product Moment correlation was used to determine relationships between variables of interest. Because of the low sample size, non-parametric tests including the Freidman test and Spearman correlation were still performed, but this did not alter the interpretation of the findings so only the results of the parametric tests are presented. Statistical significance was accepted as *P ≤* 0.05.

## Results

### Rats Body Weight Changes During the Study

**Figure 1:**
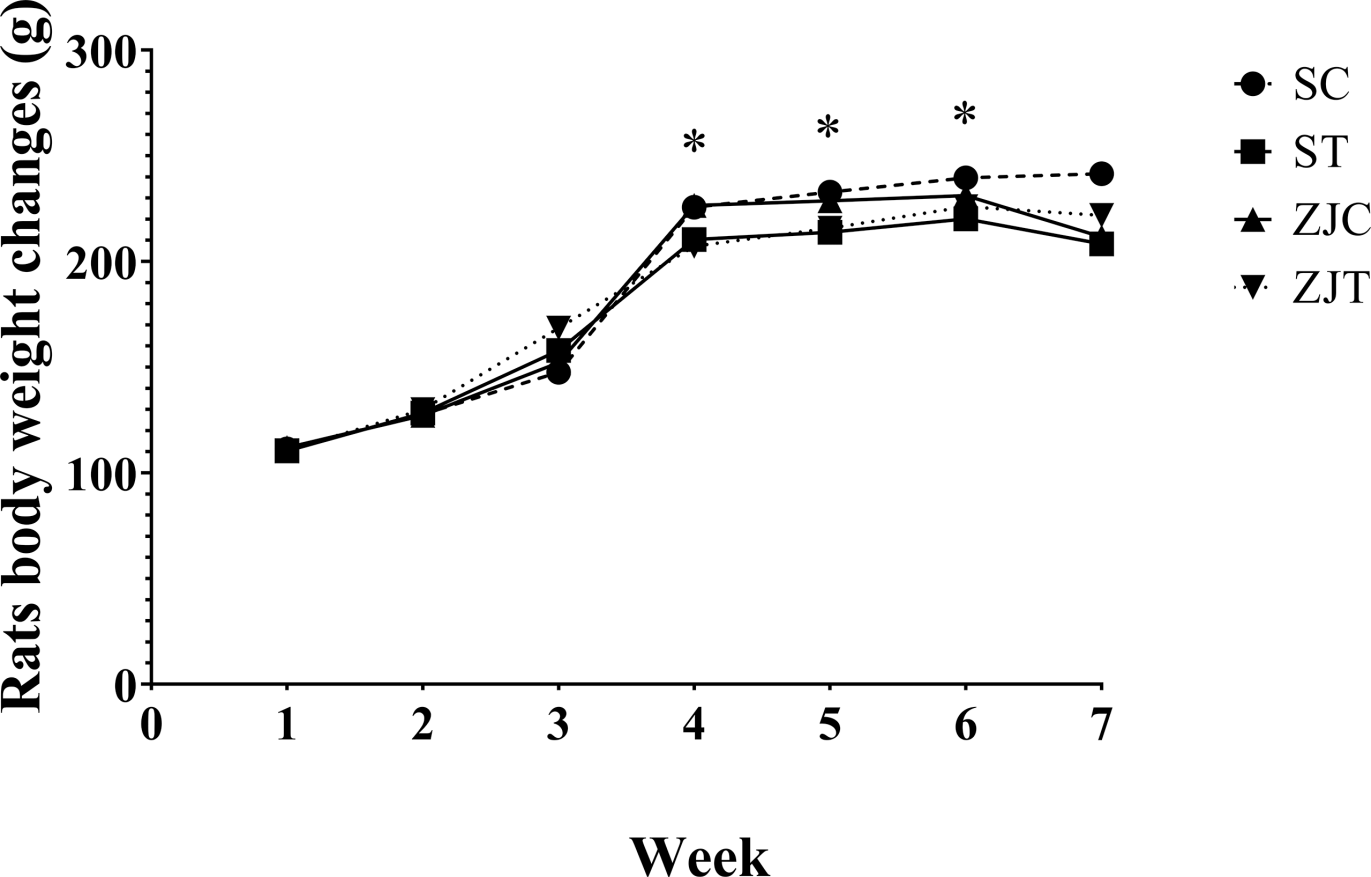
Rats body weight (g) in Saline-Control (SC), Saline-High Intensity Training (ST), *Ziziphus jujuba*-Control (ZJC), and *Ziziphus jujuba*-High Intensity Training (ZJT) groups. Data were expressed mean, P value set at < 0.05, and 7 rats per each group. * Training main effect (P=0.008 for 4^th^ week, P=0.01 for 5^th^ week and P=0.05 for 6^th^ week).

The results showed that the rats’ body weight significantly changed between groups over time (time×group effect, *F*_2.02,48.570 _= 3.493, *P*=0.038, $\eta_{\text{p}}^{2}$ = 0.127, NCP=7.070 and OPW=0.629, **Figure 1**). The rats body weight was significantly lower in trained rats compared to non-trained rats in began of 4^th^ (*F_1,24 _= *8.277, *P*=0.008, $\eta_{\text{p}}^{2}$ = 0.256, NCP=8.277 and OPW=0.788), 5^th^ (*F_1,24 _= *7.910, *P*=0.01, $\eta_{\text{p}}^{2}$ = 0.248, NCP=7.910 and OPW=0.770) and 6^th^ (*F_1,24 _= *4.105, *P*=0.05, $\eta_{\text{p}}^{2}$ = 0.146, NCP=4.105 and OPW=0.494) week (**Figure 1**). Exercise, supplementation and interaction had no significant effect on the mean of 7 repetitions of weight.

### Hypothalamus, Heart and Gastrocnemius Nesfatin-1/NUCB2 mRNA Expression

**Figure 2:**
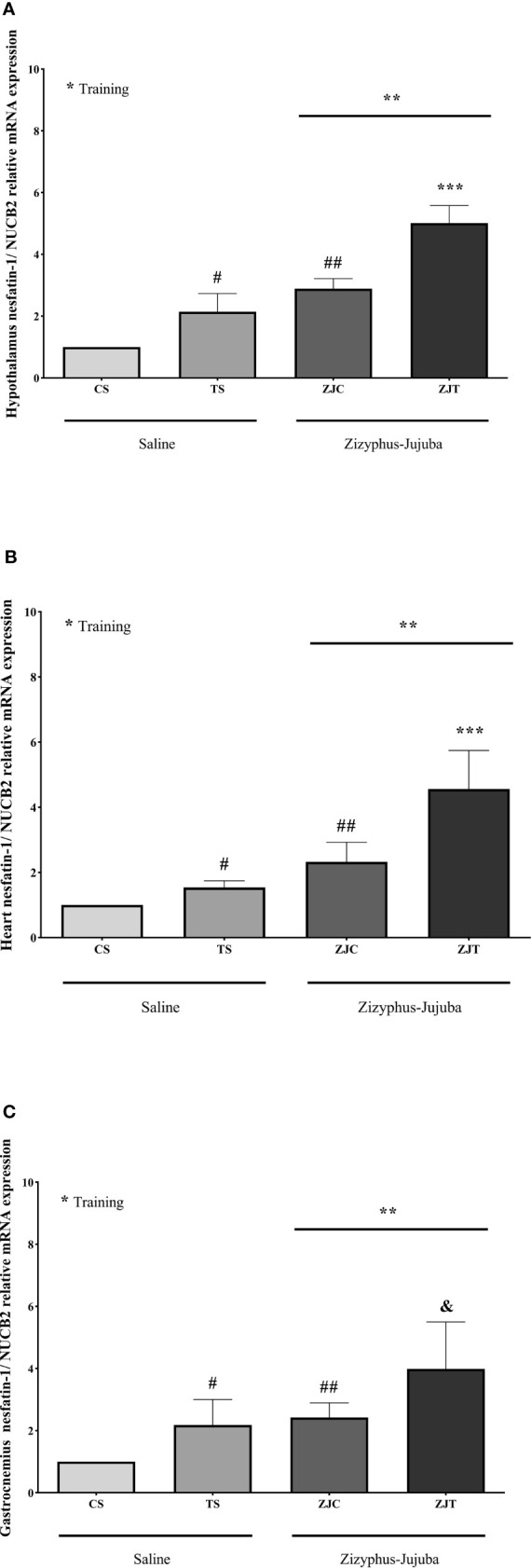
Hypothalamus **(A)**, heart **(B)** and gastrocnemius **(C)** nesfatin-1/NUCB2 gene expression than βactin in Saline-Control (SC), Saline-High Intensity Training (ST), *Ziziphus jujuba*-Control (ZJC), and *Ziziphus jujuba*-High Intensity Training (ZJT) groups. Data were expressed mean ± SD, P value set at < 0.05, and 7 rats per each group. * Training main effect (P=0.001 for hypothalamus and heart, P=0.002 for gastrocnemius), ** *Ziziphus jujuba* main effect (P=0.001 for hypothalamus, heart and gastrocnemius), *** Training and *Ziziphus jujuba* interaction (P=0.039 and P=0.035 for hypothalamus and gastrocnemius respectively), # TS than CS (P=0.001 and P=0.048 for hypothalamus and gastrocnemius respectively), ## ZJC than CS (P=0.001, P=0.02 and P=0.019 for hypothalamus, heart and gastrocnemius respectively), & ZJT than TS (P=0.001 for hypothalamus and heart, P=0.004 for gastrocnemius).

NUCB2 gene expression was significantly higher in the hypothalamus tissue (*F_1,24 _= *52.622, *P*=0.001, $\eta_{\text{p}}^{2}$ = 0.687, NCP=52.622 and OPW=1, **Figure 2A**), heart (*F_1,24 _= *13.534, *P*=0.001, $\eta_{\text{p}}^{2}$ = 0.361, NCP=13.534 and OPW=0.942, **Figure 2B**) and gastrocnemius (*F_1,24 _= *11.717, *P*=0.002, $\eta_{\text{p}}^{2}$ = 0.328, NCP=11.717 and OPW=0.907, **Figure 2C**) muscles of trained rats compared to their control. NUCB2 gene expression was significantly higher in the hypothalamus tissue (*F_1,24 _= *111.890, *P*=0.001, $\eta_{\text{p}}^{2}$ = 0.823, NCP=111.890 and OPW=1, **Figure 2A**), heart (*F_1,24_ = *33.103, *P*=0.001, $\eta_{\text{p}}^{2}$ = 0.580, NCP=33.103 and OPW=1, **Figure 2B**) and gastrocnemius (*F_1,24 _= *16.197, *P*=0.001, $\eta_{\text{p}}^{2}$ = 0.403, NCP=16.197 and OPW=0.971, **Figure 2C**) muscles of ZJ rats compared to their control. Interaction of training and ZJ showed a synergic effect regarding hypothalamus (*F_1,24 _= *4.771, *P*=0.039, $\eta_{\text{p}}^{2}$ = 0.166, NCP=4.771 and OPW=0.554, **Figure 2A**) and heart (*F_1,24 _= *5.014, *P*=0.035, $\eta_{\text{p}}^{2}$ = 0.173, NCP=5.014 and OPW=0.575, **Figure 2B**) NUCB2 gene expression. Gastrocnemius NUCB2 gene expression was significantly higher in ZJT group compared to ST and ZJ groups (P=0.004 and P=0.011, respectively, **Figure 2C**).

### Heart and Gastrocnemius Muscles Nesfatin-1 Concentration

**Figure 3:**
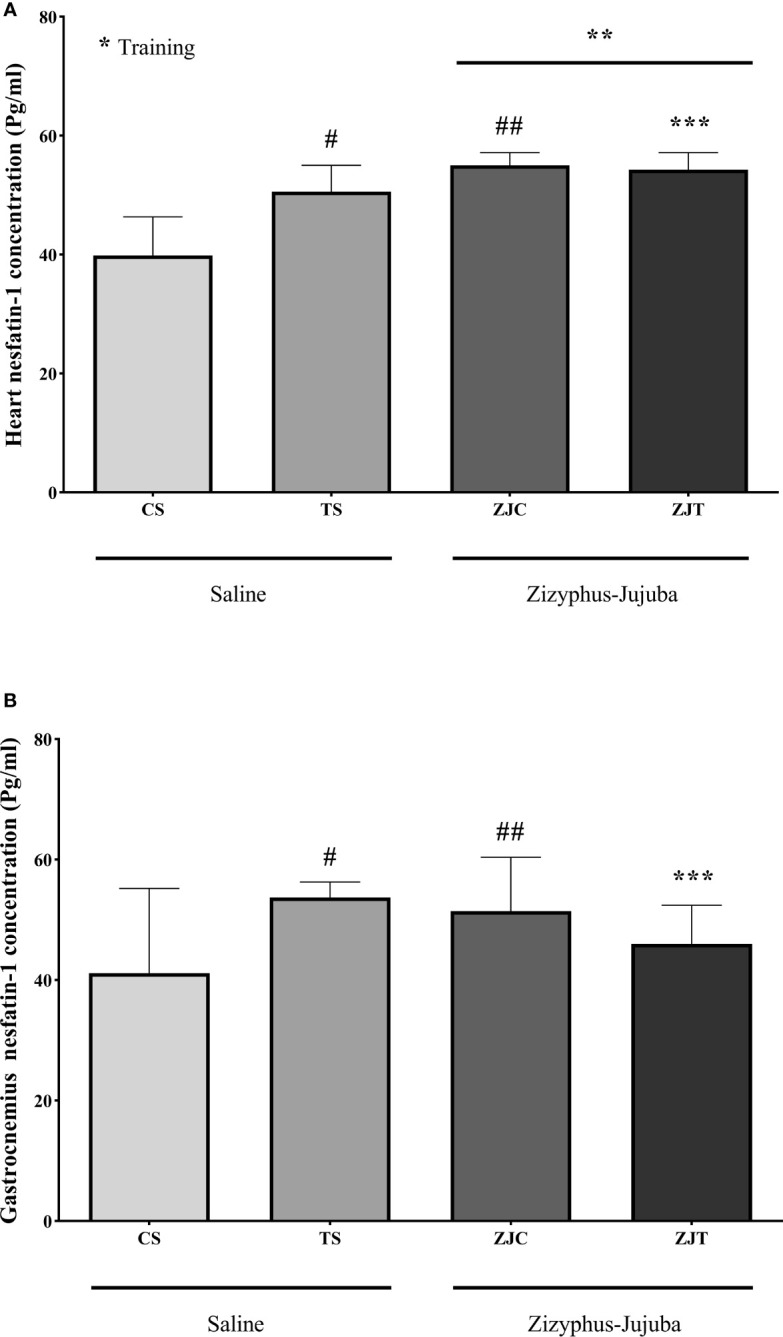
Heart **(A)** and gastrocnemius **(B)** muscles nesfatin-1 concentrations (Pg/mg) in Saline-Control (SC), Saline-High Intensity Training (ST), *Ziziphus jujuba*-Control (ZJC), and *Ziziphus jujuba*-High Intensity Training (ZJT) groups. Data were expressed mean ± SD, *P* value set at < 0.05, and 7 rats per each group. * Training main effect (P=0.005 for heart), ** *Ziziphus jujuba* main effect (P=0.001 for heart), *** Training and *Ziziphus jujuba* interaction (P=0.002 and P=0.014 for heart and gastrocnemius respectively), # TS than CS (P=0.001 and P=0.015 for heart and gastrocnemius respectively), ## ZJC than CS (P=0.001 and P=0.043 for heart and gastrocnemius respectively).

Heart nesfatin-1 concentration was significantly higher in trained rats compared to their control (*F_1,24 _= *9.375, *P*=0.005 and $\eta_{\text{p}}^{2}$ = 0.281, NCP=9.375 and OPW=0.836). The concentration of nesfatin-1 was also significantly higher in ZJ treated heart compared to saline treated rats *(F_1,24 _= *33.337*, P*=0.001 and $\eta_{\text{p}}^{2}$ = 0.581, NCP=33.337 and OPW=1). In addition, the combination of training and supplementation showed a synergetic increase in heart nesfatin-1 concentration (*F_1,24 _= *12.245*, P*=0.002 and $\eta_{\text{p}}^{2}$ = 0.338, NCP=12.245 and OPW=0.919) (**Figure 3A**). Heart and gastrocnemius muscle nesfatin-1 responded differently in that significantly higher nesfatin-1 concentrations were observed in ST (*P*=0.015) and ZJC rats (*P*=0.043), but exercise did not significantly increase or decrease nesfatin-1 concentration in ZJT (**Figure 3B**). However, in gastrocnemius muscle, nesfatin-1 concentrations were significantly higher in ST (*P*=0.015) and ZJC (*P*=0.043) when compared to SC rats and a decrease in nesfatin-1 concentration was observed in the ZJT (**Figure 3B**).

### Heart and Gastrocnemius Muscle ATP Concentration

**Figure 4:**
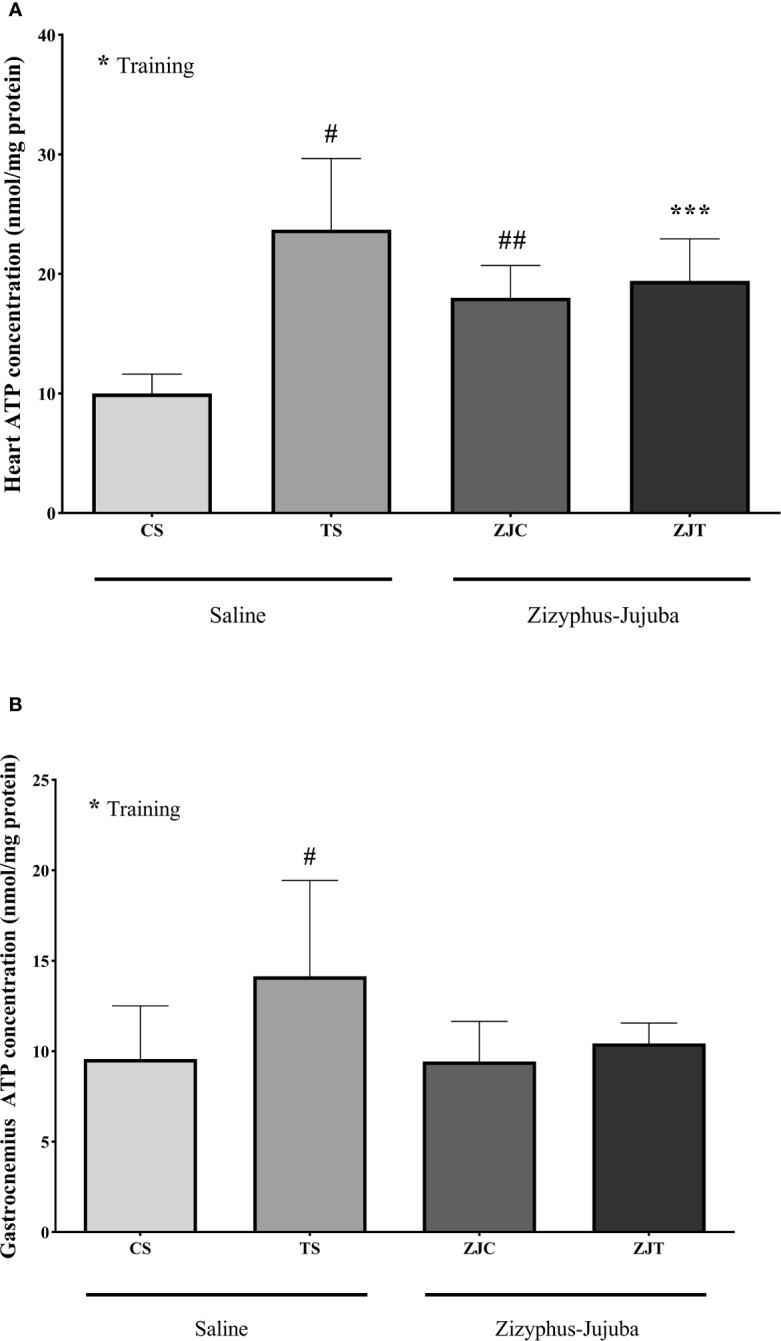
Heart **(A)** and gastrocnemius **(B)** muscles ATP concentrations (nmol/mg) in Saline-Control (SC), Saline-High Intensity Training (ST), *Ziziphus jujuba*-Control (ZJC), and *Ziziphus jujuba*-High Intensity Training (ZJT) groups. Data were expressed mean ± SD, *P* value set at < 0.05, and 7 rats per each group. * Training main effect (P=0.015 and P=0.013 for heart and gastrocnemius respectively), *** Training and *Ziziphus jujuba* interaction (P=0.05 for heart), # TS than CS (P=0.004 and P=0.01 for heart and gastrocnemius respectively), ## ZJC than CS (P=0.047 for heart).

Exercise induced significant increases in heart ATP concentration (*F_1,24 _= *6.864*, P*=0.015 and $\eta_{\text{p}}^{2}$ = 0.222, NCP=6.864 and OPW=0.710). Heart ATP was significantly higher in ST (*P*=0.004), ZJC (*P*=0.047), and ZJT (*P*=0.05) groups when compared to SC. Data also indicated that ZJT heart had significantly higher ATP concentration than SC heart (P=0.014) (**Figure 4A**). Similar changes were observed in ST, ZJC and, ZJT gastrocnemius muscle ATP concentration (**Figure 4B**). However, a higher and significant ATP concentration was only observed in the ST group (P=0.01) (**Figure 4B**).

### Heart and Gastrocnemius Muscle Glycogen Concentration

**Figure 5:**
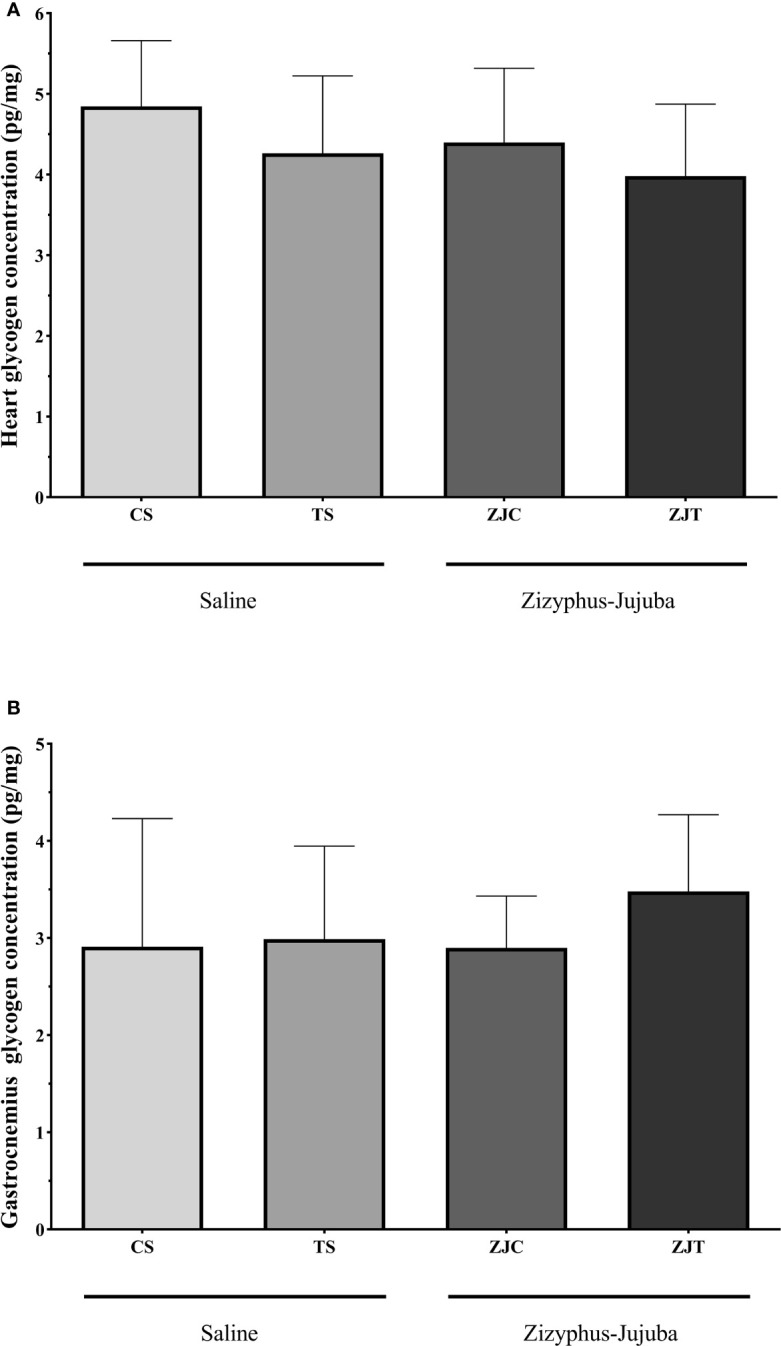
Heart **(A)** and gastrocnemius **(B)** muscles glycogen concentrations (Pg/mg) in Saline-Control (SC), Saline-High Intensity Training (ST), *Ziziphus jujuba*-Control (ZJC), and *Ziziphus jujuba*-High Intensity Training (ZJT) groups. Data were expressed mean ± SD, *P* value set at < 0.05, and 7 rats per each group.

Aerobic training had no significant effect on heart (*F_1,24 _= *1.148, *P*=0.295, $\eta_{\text{p}}^{2}$ = 0.083, NCP=2.167 and OPW=0.293, **Figure 5A**) and gastrocnemius (*F_1,24 _= *0.798, *P*=0.381, $\eta_{\text{p}}^{2}$ = 0.034, NCP=0.798 and OPW=0.137, **Figure 5B**) muscle glycogen concentration. ZJ supplementation did not significantly change heart (*F_1,24 _= *2.167, *P*=0.154, $\eta_{\text{p}}^{2}$ = 0.046, NCP=1.148 and OPW=0.177 **Figure 5A**) and gastrocnemius (*F_1,24 _= *0.416, *P*=0.546, $\eta_{\text{p}}^{2}$ = 0.018, NCP=0.416 and OPW=0.095, **Figure 5B**) glycogen concentration. There was no significant interaction between training and supplement use on heart (*F_1,24 _= *0.059, *P*=0.810, $\eta_{\text{p}}^{2}$ = 0.002, NCP=0.059 and OPW=0.056, **Figure 5A**) and gastrocnemius (*F_1,24 _= *0.471, *P*=0.499, $\eta_{\text{p}}^{2}$ = 0.020, NCP=0.471 and OPW=0.101, **Figure 5B**) glycogen concentration.

### Plasmatic Variables

**Figure 6:**
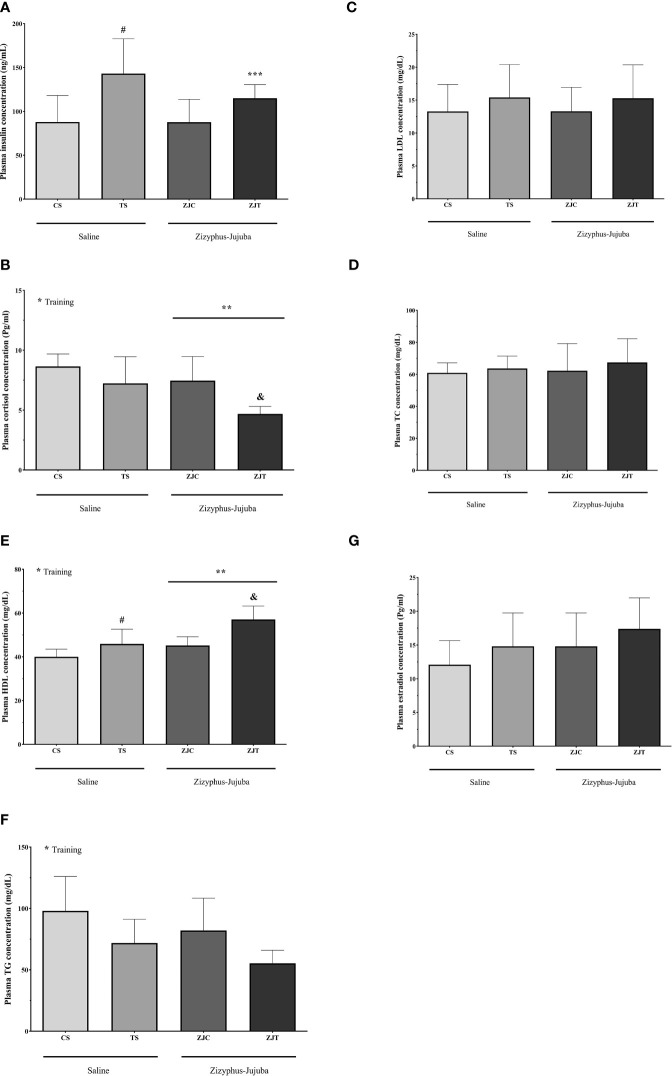
Plasma insulin (ng/ml) **(A)** cortisol (Pg/ml) **(B)**, HDL (mg/dl) **(C)**, TG (mg/dl) **(D)** LDL (mg/dl) **(E)**, TC (mg/dl) **(F)** and estradiol (Pg/ml) **(G)** concentrations in Saline-Control (SC), Saline-High Intensity Training (ST), *Ziziphus jujuba*-Control (ZJC), and *Ziziphus jujuba*-High Intensity Training (ZJT) groups. Data were expressed mean ± SD, P value set at < 0.05, and 7 rats per each group. * Training main effect (P=0.002, P=0.001 and P=0.05 for cortisol, HDL and TG, respectively, ** *Ziziphus jujuba* main effect (P=0.005 and P=0.001 for cortisol and HDL, respectively), *** Training and *Ziziphus jujuba* intraction (P=0.022 for insulin), # TS than CS (P=0.02 and P=0.046 for insulin and HDL respectively), & ZJT than TS (P=0.007 and P=0.001 for cortisol and HDL, respectively).

Plasma insulin concentration was higher in ST than CS (*P*=0.02). ZJ use led to no significant changes in plasma insulin concentration (*F_1,24 _= *0.733, *P*=0.40, $\eta_{\text{p}}^{2}$ = 0.030, NCP=0.733 and OPW=0.130). The interaction between training and supplement use showed a significant increase in plasma insulin concentration (*F_1,24 _= *5.949, *P*=0.022, $\eta_{\text{p}}^{2}$ = 0.199, NCP=5.949 and OPW=0.648) (**Figure 6A**).

Aerobic training showed a significant decrease in plasma cortisol concentration (*F_1,24 _= *11.852, *P*=0.002, ηp^2^ = 0.331, NCP=11.852 and OPW=0.911). ZJ use showed a significant decrease in plasma cortisol concentration (*F_1,24 _= *9.439, *P*=0.005 and $\eta_{\text{p}}^{2}$ = 0.282, NCP=1.264 and OPW=0.190). Plasma cortisol concentration was lower in ZJT than ST (*P*=0.007) (**Figure 6B**).

Aerobic training led to a significant increase in plasma HDL (*F_1,24 _= *19.990, *P*=0.001, $\eta_{\text{p}}^{2}$ = 0.454, NCP=19.990 and OPW=0.990, **Figure 6C**) and significant decrease in plasma TG (*F_1,24 _= *4.117, *P*=0.05, $\eta_{\text{p}}^{2}$ = 0.161, NCP=4.117 and OPW=0.495, **Figure 6D**) concentration. ZJ supplementation led to a significant increase in plasma HDL concentration (*F_1,24 _= *16.903, *P*=0.001 and $\eta_{\text{p}}^{2}$ = 0.413, NCP=19.903 and OPW=0.976). Plasma HDL concentration was higher in ZJT than ST (*P*=0.001) (**Figure 6C**).

Aerobic training had no significant effect on plasma LDL (*F_1,24 _= *1.475, *P*=0.236, $\eta_{\text{p}}^{2}$ = 0.058, NCP=1.475 and OPW=0.215, **Figure 6E**), TC (*F_1,24 _= *0.726, *P*=0.403, $\eta_{\text{p}}^{2}$ = 0.029, NCP=0.726 and OPW=0.130, **Figure 6F**) and estradiol (*F_1,24 _= *2.394, *P*=0.135, $\eta_{\text{p}}^{2}$ = 0.091, NCP=2.394 and OPW=0.318, **Figure 6G**) concentrations. ZJ supplementation had no significant effect on plasma TG (*F_1,24 _= *1.572, *P*=0.222, $\eta_{\text{p}}^{2}$ = 0.061, NCP=1.572 and OPW=0.226, **Figure 6D**), LDL (*F_1,24 _= *0.001, *P*=0.997, $\eta_{\text{p}}^{2}$ = 0.001, NCP=0.001 and OPW=0.050, **Figure 6E**), TC (*F_1,24 _= *0.297, *P*=0.591, $\eta_{\text{p}}^{2}$ = 0.012, NCP=0.297 and OPW=0.082, **Figure 6F**) and estradiol (*F_1,24 _= *2.385, *P*=0.136, $\eta_{\text{p}}^{2}$ = 0.090, NCP=2.385 and OPW=0.317, **Figure 6G**) concentrations. There was no significant interaction between training and supplement use on plasma TG (*F_1,24 _= *0.001, *P*=0.981, $\eta_{\text{p}}^{2}$ = 0.001, NCP=0.001 and OPW=0.050, **Figure 6D**), LDL (*F_1,24 _= *0.002, *P*=0.964, $\eta_{\text{p}}^{2}$ = 0.001, NCP=0.002 and OPW=0.050, **Figure 6E**), TC (*F_1,24 _= *0.072, *P*=0.790, and $\eta_{\text{p}}^{2}$ = 0.003, NCP=0.072 and OPW=0.058, **Figure 6F**) and estradiol (*F_1,24 _= *0.001, *P*=0.969 and $\eta_{\text{p}}^{2}$ = 0.001, NCP=0.001 and OPW=0.050, **Figure 6G**) concentrations.

### Relationships Between Key Variables

There were positive and significant correlations between hypothalamus gene expression with heart, nesfatin-1gene/concentration, gastrocnemius nesfatin-1 gene expression, plasma HDL and estradiol concentration, while it had negative and significant correlations with plasma cortisol and TG concentrations. Nesfatin-1 gene expression had positive and significant correlations with heart nesfatin-1 and plasma HDL concentration, while it had negative and significant correlations with heart glycogen and plasma cortisol concentration. Gastrocnemius nesfatin-1 gene expression had a positive and significant correlation with plasma HDL concentration and had negative and significant correlations with plasma cortisol and TG concentration. All correlations between key variables are shown in **Table 1**.

**Table 1:** The correlation between variables.

Variables	HypothalamusNUCB2	Heart NUCB2	GastrocnemiusNUCB2	Heart nesfatin-1	Gastrocnemius nesfatin-1	Heart ATP	Gastrocnemius ATP	Heart glycogen	Gastrocnemius glycogen	Plasma insulin	Plasma cortisol	Plasma estradiol	Plasma HDL	Plasma LDL	Plasma TG	Plasma TC
**Hypothalamus** **NUCB2**	1															
**Heart** **NUCB2**	.779**	1														
**Gastrocnemius** **NUCB2**	.742**	.536**	1													
**Heart** **nesfatin-1**	.627**	.475*	.484**	1												
**Gastrocnemius** **nesfatin-1**	.063	.037	.210	.463^*^	1											
**Heart** **ATP**	.213	.177	.179	.327	.225	1										
**Gastrocnemius** **ATP**	.061	-.015	.194	.034	.140	.266	1									
**Heart** **Glycogen**	-.269	-396*	-262	-.354	-.469^*^	-.292	.163	1								
**Gastrocnemius** **glycogen**	.180	.187	.059	.195	-.150	.188	.228	.107	1							
**Plasma** **Insulin**	.006	-.064	-.009	.156	.222	.051	.513^**^	-.260	-.041	1						
**Plasma** **Cortisol**	-.694**	-.537**	-.470*	-.530^**^	.130	-.103	.135	.179	-.133	.009	1					
**Plasma** **Estradiol**	.430*	.290	.305	.204	.044	-.041	.222	.072	.190	.252	-.286	1				
**Plasma** **HDL**	.731**	.590**	.841**	.381^*^	.002	.242	.175	-.205	.271	-.094	-.457^*^	.323	1			
**Plasma** **LDL**	.070	.074	.243	.184	.236	.218	-.009	-.314	.153	.106	-.079	.029	.320	1		
**Plasma** **TG**	-.452*	-.354	-.484**	-.431^*^	-.169	-.177	.214	.391^*^	-.249	-.101	.555^**^	-.177	-.471^*^	-.629^**^	1	
**Plasma** **TC**	.029	0.003	.238	.013	.149	.131	.102	-.215	.125	-.004	.114	.121	.442^*^	.640^**^	-.159	1

*Correlation is significant at the 0.05 level. **Correlation is significant at the 0.01 level. The rats are 28 in each variable.

## Discussion

The main findings of the present study are 1) NUCB2 gene expression was significantly higher in the hypothalamus, heart and gastrocnemius muscles of trained and ZJ supplemented rats. 2) Exercise induced significant increases in heart and gastrocnemius muscle ATP concentration. ZJ heart had significantly higher ATP concentration than Saline heart. 3) Aerobic training led to a significant increase in plasma insulin and HDL concentration and significant decrease in plasma TG and cortisol concentration. ZJ also led to a significant increase in plasma insulin and HDL concentration and significant decrease in plasma cortisol concentration. 4) A combination of exercise and ZJ showed an additive effect compared to each intervention alone on hypothalamus, heart and gastrocnemius NUCB2 gene expression, heart and gastrocnemius nesfatin-1 concentration, HDL and cortisol concentration.

Nesfatin-1 is an inhibitor of food intake, and as an anorexigenic peptide regulates several energy stress responses such as fasting/refeeding conditions or food intake (3, 15), acute exercise/training (10, 11, 26) nutrients status and obesity, and high fat diet (27). In the present study, we demonstrated that the nesfatin-1 concentration but not gene expression response differed in trained-heart and gastrocnemius muscle and this difference is likely because of tissue glycogen content at baseline. Muscle glycogen is a store of energy, but heart glycogen content is strategic storage for the hearts protection. During exercise, if lactate concentration rises, the heart uses blood lactate as a fuel, so its glycogen storage is more preserved. Nesfatin-1 is well known as a cardiac peptide which has a protective effect against some unpleasant injuries in the heart (28). Nesfatin-1 is expressed in murine and human cardiomyocytes and in the stomach (29) and has been shown to reduce during fasting leading to hypoglycemia (30). It has been shown by nesfatin-1 injection that glucose uptake increases by cardiomyocyte in human and murine cardiomyocytes *via* the translocation of GLUT-4 (29). In this study the concentration of nesfatin-1 was significantly higher in trained and supplemented than saline groups. This high nesfatin-1 concentration was accompanied with a higher ATP and glycogen concentration in Zizyphus jujuba-treated heart. It seems that glycogen is a supporter of heart ATP concentration which could help to keep a stable heart energy charge (31). The difference between the results of nesfatin-1 gene expression and its concentration may be due to the fact that gene expression has occurred but the conversion and secretion to nesfatin-1 has been less. Regardless, if gene expression occurs, it does not mean that protein conversion has occurred.

The ZJ herb contains antioxidants, amino acids and a considerable amount of vitamin trace elements with a low content of free fatty acids. Its consumption has been shown to influence appetite markers. The results also indicate that ZJ supplement increased nesfatin-1 in ZJ-treated tissue compared to the same tissues in control-saline groups. To our knowledge, no previous study has reported the effects of exercise combined with ZJ supplementation on hypothalamus, heart and gastrocnemius muscle nesfatin-1 gene/concentrations so the findings need to be confirmed or refuted.

Ghanbari-Niaki et al. reported that enhanced liver nesfatin-1 was accompanied with plasma nesfatin-1 at rest, but after running a significant decrease was observed in ZJT liver when compared with ZJC liver (9). They also found a significant reduction was observed in ST, ZJC and ZJT treated liver ATP contents when compared with saline-control liver. In addition, liver glycogen contents were not significantly higher in trained groups when compared to control groups in both treatments. Several studies have shown exercise induced significant or non-significant changes on plasma nesfatin-1 (10, 11, 32, 33). However, the information about the effect of exercise/training on nesfatin-1 mRNA gene expression and its protein content in tissues is scarce. Shirvani et al. indicate that exercise increases nesfatin-1/NUCB2gene in the liver and visceral fat which was accompanied by a significant change in plasma HDL-C (8).

Whilst speculative, there are numerous possible mechanisms causing the change in nesfatin-1 as a result of exercise. Nucleobindin-2 gene expression and nesfatin-1 protein expression in adipose tissue of murine may be regulated by tissue energy supply. In addition, fasting decreases and feeding increases murine subcutaneous adipose tissues nesfatin-1 concentration in the rat. Ramanjaneya et al. showed that nesfatin-1 concentration increased after insulin, dexamethasone, (100nM), IL-6 (20ng/mL) administration, and decreased after TNF-α administration (10ng/mL) (14). In addition, they reported that the concentration of nesfatin-1 has a negative correlation with cortisol (34). Thus, it seems that besides energy source, any change in inflammatory markers might have an impact on tissue and plasma nesfatin-1 concentrations. Nesfatin-1 is an adipokine with multiple functions, which of course would need to be evaluated for possible regulation by some inflammatory markers, which are secreted by different tissues into the circulation that might have an effect on nesfatin-1 regulation. Exploring the role of inflammatory markers on the tissues NUCB2/nesfatin-1 protein expression is warranted because it is poorly understood so further work is needed.

Tissue ATP and glycogen changes whether fed or fasted as well as food intake behavior and anorexigenic and orexigenic peptides responses have been reported by several studies. A reduction in liver ATP and liver glycogen (as a regulator of circulating glucose) was found after D-mannitol (the analogue of D-fructose), D-fructose, and ethionine injection (35–38). The injection of ethionine as an analogue of methioine has shown to reduce liver ATP and glycogen profoundly and increase food intake in ethionine treated rats (39). However, phosphate loading has been shown to prevent D-mannito-induced ATP and glycogen reduction by restoring trapped phosphate in tissues (37, 39). Ghanbari-Niaki et al. observed a profound reduction in liver ATP and glycogen after an acute ethionine injection in rats (38) which has been accompanied with a higher plasma ghrelin concentration in rats. These findings suggests cellular energy deficiency, particularly a profound reduction in tissues, especially in the liver could stimulate food intake by enhancing some appetite peptide whose involved in food intake and energy regulation and balance.

In the gastrocnemius muscle, nesfatin-1 concentration was increased in the ST group but reduced in the ZJT group. This differing response is likely due to a significant elevation in heart ATP content in both ST and ZJ supplemented rats. However, the magnitude of ATP change was not high enough to reach significance in trained muscle, supplemented with ZJ. These difference may be due to biochemical characteristics of muscle fibers.

To the authors knowledge no other study has examined the effects of ZJ supplement on HDL concentrations. We found that trained rats had higher plasma HDL concentrations compared to control groups and this increase was also augmented by ZJ supplementation. Several studies showed that exercise training induced an increase in plasma HDL concentration and functionality (40). Both aerobic exercise training and ZJ treatment induced significant increase in fundus tissue nesfatin-1 and plasma concentration of HDL (41).

Significant and lower cortisol concentration was observed in ZJ groups when compared with saline groups. The present study is the first study to examine the effects of ZJ supplement on cortisol thus the mechanisms by which the decrease occurs is unclear and remains to be elucidated. ZJ supplement is high in carbohydrate and a previous study showed that a high CHO diet induced a decrease in cortisol (42). Supplementation with 7 % sugar solutions during endurance training causes a lowering of post-exercise concentration of hydrocortisone in the blood while the consumption of liquids containing up to 1.5 % carbohydrate or maltodextrin solutions (8 %) during training has no effect on cortisol (43).

Insulin concentration was higher in the exercise groups than in the non-exercise groups. Exercise training had no effect on serum insulin in obese rats (44) and has been shown to decrease (37) or increase concentration in rats (45). The difference in studies is likely due to the intensity of exercise with high intensity exercise stimulating a rise.

Our study has a number of strengths including we investigate the effect of Ziziphus jujuba extract on heart and gastrocnemius muscle nesfatin-1 for the first time as well as the combination of aerobic exercise training and Ziziphus jujuba extract on heart and gastrocnemius muscle nesfatin-1 and related variables. All experimental trials and methods were meticulously conducted using standardized operating procedures and the associated biochemistry was undertaken by experts, using assays by reputable companies with acceptable correlation coefficients.

The limitations of our study are that we did not measure inflammatory factors such as TNFα or IL-6. Neither did we measure protein expression of NUCB2 (by Western blot method) in which such findings would have allowed us to comment other potential influencers of Nestafin-1. Finally, our results cannot be generalized to humans which should be a future line of enquiry. A number of further research questions have therefore arisen as a result of this work including What is the effect of these interventions alone or in combination on protein expression of NUCB2 and inflammatory factors.

## Conclusions

The present study showed an increase in tissue nesfatin-1 gene/concentration following high-intensity aerobic training and ZJ in rats. This highlights the beneficial effects of this type of exercise and supplement on a key neuropeptide involved in metabolic regulation and feeding behavior. Ziziphus jujuba supplementation has positive effects on multiple outcomes including an increase plasma insulin and reduction in cortisol concentration and is recommended with exercise to improve the health of rats. Our novel findings need to be confirmed or refuted and studies in humans are warranted.

## Data Availability Statement

The original contributions presented in the study are included in the article/supplementary material. Further inquiries can be directed to the corresponding author.

## Ethics Statement

The animal study was reviewed and approved by University of Mazandaran.

## Author Contributions

AG-N conceived and designed this study. FH, BT, and SR-A collected the data. SR-A and DB wrote the manuscript. All authors read and approved the final version of the manuscript.

## Conflict of Interest

The authors declare that the research was conducted in the absence of any commercial or financial relationships that could be construed as a potential conflict of interest.

## Publisher’s Note

All claims expressed in this article are solely those of the authors and do not necessarily represent those of their affiliated organizations, or those of the publisher, the editors and the reviewers. Any product that may be evaluated in this article, or claim that may be made by its manufacturer, is not guaranteed or endorsed by the publisher.
